# The complete mitogenome of Bagrid catfish *Chrysichthys nigrodigitatus* (Siluriformes: Claroteidae)

**DOI:** 10.1080/23802359.2018.1532341

**Published:** 2018-10-25

**Authors:** Nack-Keun Kim, Fantong Zealous Gietbong, Sapto Andriyono, Ah Ran Kim, Hyun-Woo Kim

**Affiliations:** a Interdisciplinary Program of Biomedical, Mechanical and Electrical Engineering, Pukyong National University, Busan, Republic of Korea;; b Department of Marine Biology, Pukyong National University, Busan, Republic of Korea;; c KOICA-PKNU International Graduate Program of Fisheries Science, Graduate School of Global Fisheries, Pukyong National University, Busan, Republic of Korea;; d Department of Marine, Fisheries and Marine Faculty, Universitas Airlangga C Campus Jl. Mulyorejo Surabaya East Java, Indonesia

**Keywords:** *Chrysichthys nigrodigitatus*, mitochondrial genome, catfish, Cameroon, Africa

## Abstract

We here report the complete mitochondrial genome of *Chrysichthys nigrodigitatus*, which is 16,514 bp in length. Mitogenome of *C. nigrodigitatus* showed the conserved 13 protein-coding genes, 22 tRNA genes, two rRNA genes, and two noncoding regions including the light-strand replication origin (OL) and a putative control region (CR). All tRNA genes were predicted to fold into the typical cloverleaf secondary structures with the typical base-pairing except for tRNA-Ser(AGC). Phylogenetic analysis with currently known complete mitogenome sequences in Siluriformes showed that *C. nigrodigitatus* is most closely related to *Auchenoglanis occidentalis* forming a family Claroteidae cluster.

Bagrid catfish, *Chrysichthys nigrodigitatus,* is one of highly valulable fresh-water fish resources in West African countries including Nigeria, Senegal, and Cameroon (Azeroual et al. 2010; Andem et al. 2013). Due to the recent increased demands for the species, the sustainable production of *C. nigrodigitatus* is highly required both by aquaculture without the adverse effects on the aquatic ecosystem (Kareem et al. 2015) and by the scientific management of native populations (Abu and Agarin 2016). However, the limited genetic inforamtion of *C. nigrodigitatus* is now a major weakness for their effective managements.

In this study, total mitochondrial genome of *C. nigrodigitatus* was determined by the next generation sequencing (NGS) platform. The specimen was collected from the coastal water of Bamusso (4°00′09″N 09°14′40″E) on October 2017, Cameroon. Species identification and storage was conducted by Fisheries and Oceanographic Research Station (IRAD Batoke), Cameroon. COI region of the specimen showed 100% identity to *C. nigrodigitatus* (GenBank no. HG803416). Genomic DNA extraction was done by Accuprep^®^ Genomic DNA Extraction Kit (Bioneer, Korea) kit following the manufacturer’s protocol. Two large mitochondrial PCR products (11 kb and 6 kb) were obtained by two sequence-specific primer sets, which targeted COX1 and ITS and they were further fragmented into smaller-sized DNAs (∼350 bp) by Covaris M220 Focused-Ultrasonicator (Covaris Inc., Woburn, MA). A library was constructed by TruSeq^®^ RNA library preparation kit V2 (Illumina, San Diego, CA) and sequencing was performed by MiSeq sequencer (2 × 300 bp). Full mitochondrial sequences were assembled by Geneious v 11.0.2 and phylogentic analysis was conducted by MEGA7 with minimum evolution arlgorithm (Kumar et al. 2016). The secondary structure of tRNA genes were predicted using ARWEN (Laslett and Canbäck 2008).

The circular mitogenome of *C. nigrodigitatus* (GenBank no. MH709123) was 16,514 bp in length in which 13 protein-coding genes, 22 tRNAs, two ribosomal RNAs, and two non-coding regions (*OL* and *D-Loop*). All the protein-coding genes utilized ATG as the start codon, except for COI (GTG) and ND6 (ATT) genes. Seven genes (ND2, COII, ATP6, COIII, ND3, ND4, and Cyt b) showed the incomplete stop codons (TA and T). Among these protein-coding genes, only ND6 genes were encoded on L-strand, while the 12 protein-coding remaining were encoded on H-strand position. All tRNA genes were predicted to fold into the typical cloverleaf secondary structures with the typical base-pairing except for tRNA-Ser^(AGC)^. The two commonly found noncoding regions among verterbrates, *D-loop* (882 bp) and *OL* (31 bp) was located between tRNA-Pro and tRNA Phe, and tRNA-Asn and tRNA-Cys, respectively. The phylogenetic analysis showed that *C. nigrodigitatus* is the most closely related to *Auchenoglanis ocidentalis* (GenBank no. NC015809) with 84% sequence identity forming a family claroteidae cluster (Figure 1). More mitogenome information in the claroteidae should be supplemented for the clear evolutional relationship of *C. nigrodigitatus* within the family.

**Figure 1.**
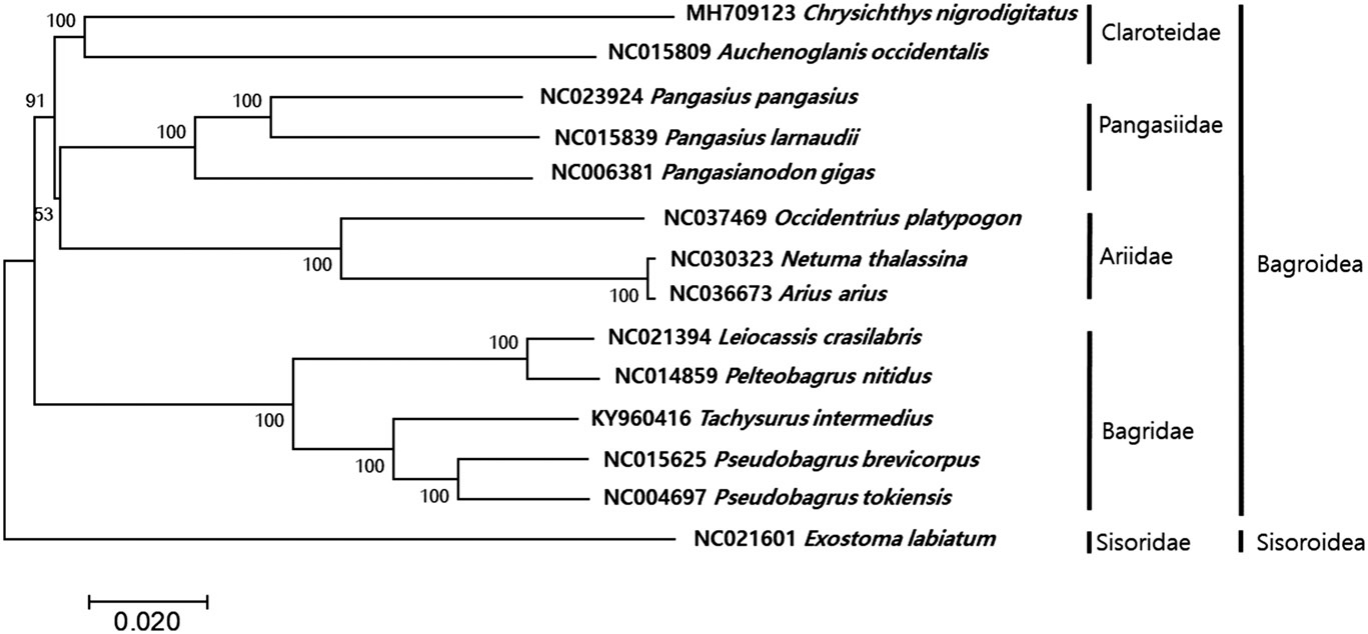
Phylogenetic tree of *Chrysichthys nigrodigitatus* within Siluriformes Order. Phlyogenetic tree of complete genome was constructed by MEGA7 software with minimum evolution (ME) algorithm with 1000 boothstrap replications. GenBank accession numbers were shown followed by each scientific name. The sequence data for phylogenetic analyses used in this study were as follows: *Chrysichthys nigrodigitatus* (MH709123), *Auchenoglanis occidentalis* (NC015809), *Natuma thalassina* (NC030323), *Arius arius* (NC036673), *Occidentarius platypogon* (NC037469), *Pangasianodon gigas* (NC006381), *Pangasius larnaudi* (NC015839), *Pangasius pangasius* (NC023924), *Leiocassis crassilabris* (NC021394), *Pelteobagrus nitidus* (NC014859), *Tachysurus intermedius* (KY962416), *Pseudobagrus brevicorpus* (NC015625) *Pseudobagrus tokiensis* (NC004697), and *Exostoma labiatum* (NC021601).
